# IGHG1 promotes malignant progression in breast cancer cells through the regulation of AKT and VEGF signaling

**DOI:** 10.17305/bb.2022.8508

**Published:** 2023-08-01

**Authors:** Yong Zhang, Xueying Fang, Yan Sun

**Affiliations:** 1 Department of Surgery, Guang’anmen Hospital, China Academy of Chinese Medical Sciences, Beijing, China; 2 Department of Oncology, Beijing Puxiang Traditional Chinese Medicine Cancer Hospital, Beijing, China

**Keywords:** Breast cancer, vascular endothelial growth factor (VEGF), AKT, immunoglobulin heavy constant chain gamma 1 (IGHG1)

## Abstract

Immunoglobulin heavy constant chain gamma 1 (IGHG1) is highly expressed in a variety of cancers and is considered an emerging prognostic marker. Overexpression of IGHG1 in breast cancer tissues has also been demonstrated, but an in-depth analysis of its role in disease progression has not been explored. In this study, we used a range of molecular and cell-based assays to show that increased expression of IGHG1 in breast cancer cells activates AKT and vascular endothelial growth factor (VEGF) signaling, leading to enhanced cell proliferation, invasion, and angiogenesis. We further showed that *IGHG1*-silencing can suppress the neoplastic characteristics of breast cancer cells in vitro and suppresses tumor growth in nude mice. These data reveal a key role of IGHG1 in the malignant progression of breast cancer cells and highlight its potential as a prognostic marker and therapeutic target to control metastasis and angiogenesis in malignant breast tissue.

## Introduction

Breast cancer is the most prevalent malignancy in females [1]. Progesterone, estrogen, and HER2 receptors are often used to characterize the disease [2]. Malignancies that lack the expression of these three receptors are referred to as triple negative breast cancer and are more difficult to diagnose and contribute to increased mortality [3]. There is an increasing need to identify effective prognostic markers and therapeutic targets to correlate with the treatment and progression of triple negative breast cancer malignancies.

The presence of plasma cell infiltrates and elevated expression of immunoglobulin G (IgG) in various cancers has been reported [4, 5]. Specifically, the upregulation of IgG containing immunoglobulin heavy constant chain gamma 1 (IGHG1) is strongly associated with various malignancies, including colorectal cancer, gastric cancer, prostate cancer, papillary thyroid carcinoma, leukemia, ovarian cancer, and breast cancer [6–8]. The molecular mechanisms exploited by IGHG1 during malignant progression have been demonstrated in various studies [8, 9]. In colorectal cancer, the upregulation of IGHG1 contributes to increased cellular proliferation [7, 10]. MEK-FECH signaling is upregulated in IGHG1-overexpressing colorectal carcinomas [11]. IGHG1 overexpression has also been reported for the induction of epithelial to mesenchymal cell transmission (EMT) in gastric cancer via tumor growth factor beta (TGF-β)/SMAD3 signaling, whilst AKT/glycogen synthase kinase 3 beta (GSK-3β)/β-catenin pathway is also highly active [12]. In prostate cancer, the inhibition of IGHG1 is linked to the suppression of MEK/ERK/c-Myc signaling, leading to reduced malignant growth [9].

The upregulation of IGHG1 has also been reported in breast cancer [9, 12], but its association with cell proliferation and disease progression at the molecular level has not been established. Further validation studies are also required to confirm the association of IGHG1 with proliferation, invasion, and angiogenesis in heterogeneous breast cancer tissues that show poor prognosis.

In this study, we show that IGHG1 is upregulated in breast cancer tissue and is associated with poor prognosis. We further explore the role of the IGHG1 in breast cancer progression and explore its potential as a future therapeutic candidate.

## Material and methods

### GEPIA

The Gene Expression Profiling Interactive Analysis (GEPIA) database (http://gepia.cancer-pku.cn/) (07.03.2022) was used to analyze the expression of *IGHG1* between 1085 breast cancer tissues and 295 normal tissues in The Cancer Genome Atlas (TCGA) RNA-seq raw data.

### Tissue samples

A total of 70 breast cancer tissue samples were collected from the China–Japan Friendship Hospital. The ages of the patients ranged from 40 to 70 years old. Full consent was provided by all study participants. The inclusion criteria were as follows: 1) newly diagnosed with breast cancer between 2000 and 2018 and 2) diagnosis and treatment criteria followed in accordance with hospital cancer treatment guidelines. Exclusion criteria were as follows: 1) patients with an absence of cancer-staging or 2) lost to follow-up. Tumor tissues were stored in liquid nitrogen prior to analysis.

### Cell lines

Breast cancer cell lines (MCF-7, T-47D, MDB-MB-231, and BT-549) and the normal epithelial breast cell line (MCF10A) were purchased from the American Tissue Culture Collection (ATCC, Manassas, Virginia, USA). MCF10A cells were maintained in DMEM/F12 1:1 (Gibco, USA). Breast cancer cells were cultured in DMEM medium (Sigma Aldrich, USA) containing 10% fetal bovine serum (FBS, Gibco, USA) in a humidified CO_2_ incubator at 37 ^∘^C.

### Constructs and transfection

The expression vector pcDNA 3.1 was designed by Invitrogen in *Escherichia coli K12* DH10B™ T1R. The full length of the vector was 6248 bp. The *IGHG1* gene was constructed and verified using forward primer (5’-3’) GTTTTCGTCGTTGCCCTTTTAAG and reverse primer ACCCACTGAATGAGAATCCAGAG. *IGHG1* was transfected into MCF-7 and T47D cells using Lipofectamine 3000, Thermofisher Scientific (cat: L3000001) at 70%–80% confluency. Three *IGHG1* sh-RNA encoding constructs, namely, sh-*IGHG1*#1, sh-*IGHG1*#2, and sh-*IGHG1*#3, were a gift from the Quanzhou First Hospital Affiliated to Fujian Medical University and transfected into MDA-MB-231 and BT-549 cells for silencing studies.

### Immunohistochemistry

Clinical breast cancer tissues were analyzed by immunohistrochemistry (IHC) to analyze IGHG1 expression as previously described [11]. Deparaffinized sections were subjected to antigen retrieval by microwave heating in 10 mM citrate buffer. Fixation was performed using paraformaldehyde. Cells were permeabilized, blocked, and labeled with anti-IGHG1 (1:1000, ab109489, Abcam, Shanghai, China). Immunostained tissues were analyzed using the HRP detection system.

### Real-time polymerase chain reaction

Breast cancer tissues and cell lines were subjected to RT-PCR for *IGHG1* expression analysis. RNA extractions were performed using ThermoFisher PureLink™ RNA Mini Kit (cat: 12183020, ThermoFischer, USA). cDNA synthesis was performed using Thermo Scientific Rever Aid Kit (Cat: K1621, ThermoFischer, USA). Extracted RNA samples were immediately processed for cDNA synthesis. *IGHG1* primers were as follows: forward: *IGHG1*, 5’-ACTCCGACGGCTCCTTCTTC-3’ (forward) and *IGHG1*, 5’-TTCTGCGTGTAGTGG TTGTGC-3’ (reverse). SYBR green (TOYOBO, Japan) was used for qPCR analysis. mRNA expression was assessed using the 2^−ΔΔCt^ method. *GAPDH* was used as an internal control.

### Western blotting

The expression of IGHG1, AKT, p-AKT, and vascular endothelial growth factor (VEGF) were analyzed in cell lines and breast cancer tissues [13]. Cells were lysed and protein levels were assessed via BCA assays (ThermoFischer Scientific cat: 23225). Proteins were resolved using 10% sodium dodecyl sulphate polyacrylamide gel electrophoresis (SDS-PAGE) and transferred to polyvinylidene difluoride (PVDF) membranes (Beyotime, Shanghai, China). Membranes were blocked in Tris buffer containing 5% skimmed milk and probed with the following primary antibodies: anti-IGHG1 (ab 283421, Abcam, China), anti-AKT (ab 8805), anti-p-AKT (ab 81283), anti-β-actin (ab 8227), and anti-VEGF (ab 53465) at 4 ^∘^C followed by labeling with HRP-conjugated goat anti-rabbit antibodies (1:1,000; ab 7090) for 2 h at room temperature. Bands were visualized using chemiluminescence detection kits (Thermo Fisher Scientific, Inc., USA).

### Cell proliferation, invasion, and angiogenesis assays

EdU assays were performed using commercial EdU cell proliferation kits (Abcam, ab 219801) for 2 h. Cells were washed with PBS, DAPI-stained, and imaged on an inverted fluorescent microscope.

Transwell assays were performed as previously described [9]. MDA-MB-231 and BT-549 cells were transfected with control vector and *IGHG1* constructs and 60,000 cells were seeded into the upper chambers of transwell inserts pre-coated with Matrigel (BD Biosciences, MD, USA). The lower chamber was supplemented with complete medium. After 48 h, cells in the lower chambers were fixed and stained using crystal violet. In each sample, 10 randomly chosen fields were selected for counting penetrating cells.

Endothelial cell tube formation assays were performed using human umbilical vein endothelial cells (HUVEC) as previously described [14]. Briefly, conditioned media was collected from MDA-MB-231 and BT-549 cells. The HUVECs were serum starved for 24 h then incubated with basement membrane extract (BME) (10 mg/mL) or conditioned media for 6 h. Tube formation was observed under an inverted microscope.

### In vivo studies

Male nude C57BL/6J mice (6–8 weeks-old, *n* ═ 6) were purchased from the Jackson Laboratory, Bar Harbor, ME, USA. C57BL/6J is a line of laboratory mice. All experimental procedures were performed and approved by the ethics committee of Beijing Viewsolid Biotechnology Co. LTD (VS212601451) and performed in accordance with the relevant guidelines and regulations. Two groups (control and *IGHG1*-knockdown), each containing three mice, were formed. The control group was subcutaneously injected with 1 million MDA-MB-231 cells transfected with sh-NC vector. The *IGHG1*-knockdown group was transfected with sh-*IGHG1*. Five weeks later, tumor tissues were harvested from mice sacrificed using ketamine (50 mg/kg).

**Figure 1.**
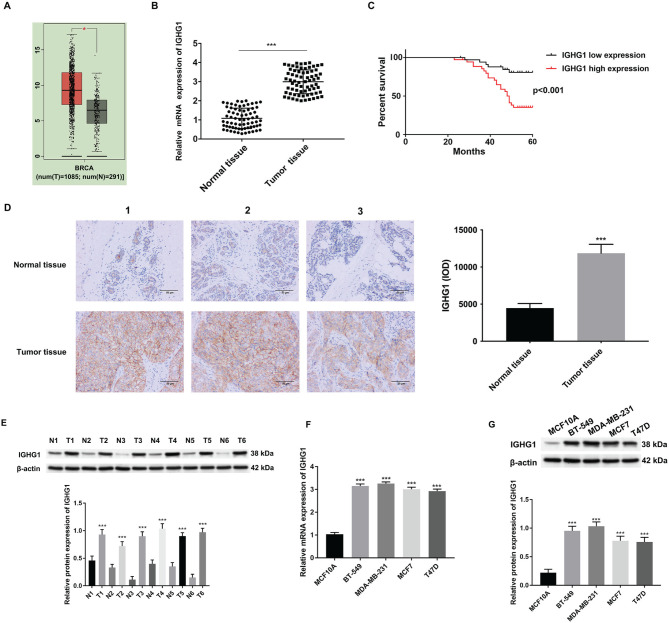
**IGHG1 is highly expressed in breast cancer tissues and cell lines and is associated with poor prognosis**. (A) GEPIA online analysis of TCGA database found that *IGHG1* was highly expressed in breast cancer; (B) Expression of *IGHG1* in 70 pairs of breast cancer and corresponding adjacent tissues detected by qRT-PCR; (C) Kaplan–Meier survival curves were used to evaluate the overall survival rate of low and high *IGHG1* expression groups; (D) IGHG1 expression in three representative breast cancer and adjacent tissues detected by immunohistochemistry; (E and F) Protein and mRNA expression level of IGHG1 in breast cancer cell lines (BT-549, MDA-MB-231, MCF7, and T47D) and normal mammary epithelial MCF10A cells detected by qRT-PCR; (G) Protein expression of IGHG1 in breast cancer cell lines (BT-549, MDA-MB-231, MCF7, and T47D) and normal breast epithelial cell MCF10A cells. IGHG1: Immunoglobulin heavy constant chain gamma 1; GEPIA: Gene Expression Profiling Interactive Analysis; TCGA: The Cancer Genome Atlas.

### Ethical statement

The study was approved by the Ethics Committee of China–Japan Friendship Hospital (2016-SDTS-04) and conducted according to the Declaration of Helsinki.

### Statistical analysis

Western blot analysis was performed using Image J (National Institutes of Health, USA). Data were analyzed using Statistical Product and Service Solutions (SPSS) 21.0 statistical software (IBM Corp., Armonk, NY, USA). Experiments were run in triplicate and represent the mean ± standard deviation (SD). Single group comparisons were performed using *t*-test. Multiple group comparisons were performed using a one-way analysis of variance (ANOVA). *P* < 0.05 was considered a significant difference.

## Results

### IGHG1 is overexpressed in breast cancer tissue

Online GEPIA analysis of TCGA database suggested that *IGHG1* levels are significantly higher in breast cancer tissue compared to normal breast tissue (Figure 1A). To study this, the mRNA expression of *IGHG1* was assessed in 70 breast cancer tissue samples and adjacent normal tissues. qRT-PCR analysis revealed remarkably higher expression of *IGHG1* mRNA in breast cancer tissue compared to adjacent normal tissue (Figure 1B). Based on the cutoff value of the median expression of *IGHG1* in breast cancer tissues (Figure 1B), 70 patients were assigned to the low *IGHG1* expression group and 35 to the high expression group. The overall survival rate of these two groups was calculated using Kaplan–Meier survival curves. The high *IGHG1* group showed a significant correlation to poor prognosis and survival (Figure 1C).

A total of three representative breast cancer tissues and adjacent normal tissues were further evaluated for IGHG1 expression using IHC. High IGHG1 expression levels in these tissues compared to adjacent normal tissue were observed, consistent with the qRT-PCR analyses (Figure 1D). qRT-PCR and western blot analysis of four breast cancer cell lines MCF-7, MDA-MB-231, T47D, and BT-549 revealed considerably higher IGHG1 mRNA and protein expression compared to the normal epithelial cell line MCF-10A (Figure 1E–1G). As shown in Table 1, no significant difference between the high and low IGHG1 expression groups with regard to age, tumor number, estrogen receptors, and HER2 was observed. High IGHG1 expression was, however, significantly associated with tumor diameter, pathological stage, pathological grade, and progesterone receptors (*P* < 0.05).

**Table 1:** Comparison of characteristics of the breast cancer patients with high and low IGHG1 expression

**Parameter**	***N***	**Low IGHG1 (*N* ═ 35)**	**High IGHG1 (*N* ═ 35)**	***P* value**
Age (years)				
≥60	26	8	18	0.352
<60	44	14	30	
Tumor diameter (cm)				
≥2	52	18	34	0.018
<2	18	12	6	
Tumor characteristics				
Unicentric	38	18	20	0.276
Multicentric	32	14	18	
T stage				
T1	28	10	18	0.037
T2–T4	42	15	27	
Pathological grade				
G1	33	10	23	0.002
G2–G3	37	14	23	
PR				
+	31	13	18	0.001
−	39	11	28	
ER				
+	30	14	16	0.258
−	40	18	22	
HER2				
+	42	22	20	0.288
−	28	12	16	

IGHG1: Immunoglobulin heavy constant chain gamma 1; PR: Progesterone receptor; ER: Estrogen receptor.

### Elevated IGHG1 levels promote cell proliferation, invasion, and angiogenesis

**Figure 2.**
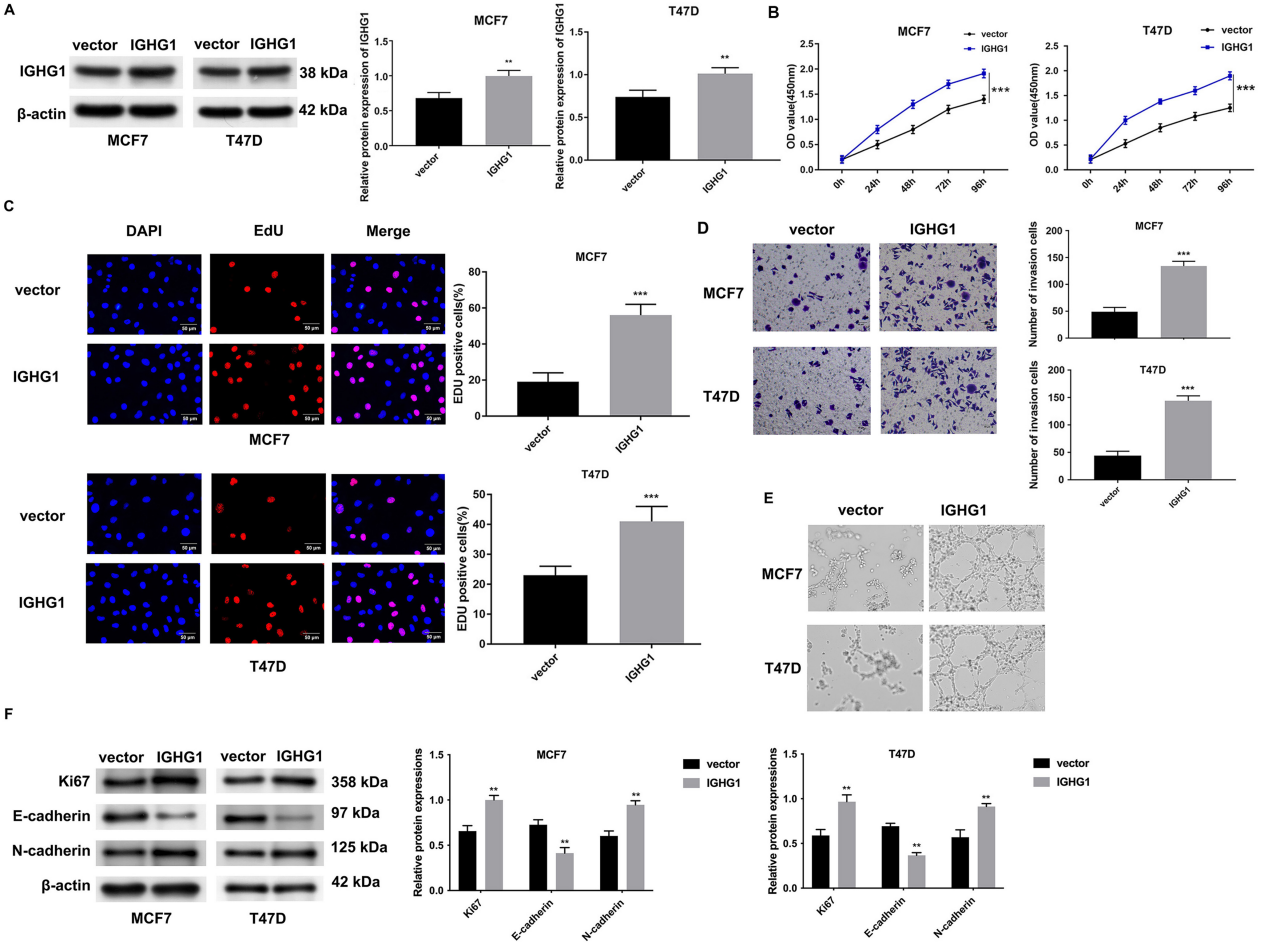
**Overexpression of IGHG1 promotes the proliferation, invasion, and angiogenesis of breast cancer cells.** (A) Western blot analysis of IGHG1 overexpression; (B) CCK8 analysis of MCF7 and T47D cells at 0, 24, 48, 72, and 96 h post-transfection; (C) Positive rates of EdU staining in transfected cells; (D) Transwell-invasion assays were used to detect the invasive ability of MCF7 and T47D cells in the different groups (vector, *IGHG1*); (E) Tube formation assays in MCF7 and T47D cells following co-culture with HUVEC supernatants; (F) Ki67, E-cadherin, and N-cadherin expression in MCF7 and T47D cells were detected by western blotting. *GAPDH* was probed as an internal control. IGHG1: Immunoglobulin heavy constant chain gamma 1; CCK8: Cell Counting Kit 8; HUVEC: Human umbilical vein endothelial cells.

IGHG1 expression was notably higher in *IGHG1*-transfected MCF-7 and T47D cells (Figure 2A). The proliferation of both transfected cell lines was measured at 0, 24, 48, 72, and 96 h using Cell Counting Kit 8 (CCK8) assays. The viability of MCF7 and T47D cells increased following the overexpression of IGHG1 (Figure 2B). EdU staining showed that IGHG1 expression also increased cell proliferation (Figure 2C). A 2-fold increase in cell migration and angiogenesis was also observed in IGHG1-overexpressing cells (Figure 2D and 2E). Ki67 and N-cadherin expression were higher in IGHG1-expressing cells, whilst E-cadherin expression was downregulated (Figure 2F).

### IGHG1 inhibition suppresses the proliferation and invasion of cancer cells

**Figure 3.**
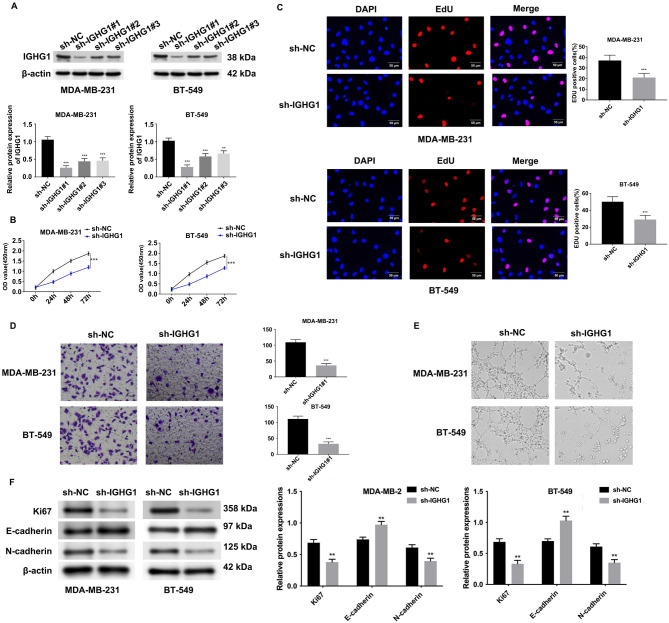
**Knockdown of *IGHG1* inhibits the proliferation, invasion, and angiogenesis of breast cancer cells.** (A) Western blot detection of sh-*IGHG1* knockdown (sh-NC, sh-*IGHG1*#1, sh-*IGHG1*#2, and sh-*IGHG1*#3) of MDA-MB-231 and BT-549 cells; (B) CCK8 analysis of sh-NC and sh-*IGHG1* groups; (C) EdU assays; (D) Transwell-invasion assays to detect the invasive ability of MDA-MB-231 and BT-549 cells in sh-NC and sh-*IGHG1* groups; (E) Tube formation assays; (F) Ki67, E-cadherin, and N-cadherin expression in sh-NC and sh-*IGHG1* groups. IGHG1: Immunoglobulin heavy constant chain gamma 1; CCK8: Cell Counting Kit 8.

To further strengthen the association of IGHG1 with breast cancer severity, *IGHG1*-specific shRNA constructs were used to silence *IGHG1* expression. Three shRNA constructs were verified by western blot analysis (Figure 3A). The sh*-IGHG1*#1 group showed a ∼3-fold reduction in *IGHG1* expression and was selected for further experiments. CCK-8 and EdU-based DNA synthesis analyses showed a ∼40% reduction in cell growth rates (Figure 3B and 3C). Transwell invasion assays of sh-*IGHG1*#1-transfected MDA-MB-231 and BT-549 cells were also significantly reduced (Figure 3D), along with a reduction in angiogenesis (Figure 3E). Ki67 and N-cadherin expression decreased, whilst E-cadherin expression increased following *IGHG1*-silencing (Figure 3F). Together, these data confirmed a key role for IGHG1 during breast cancer cell progression.

**Figure 4.**
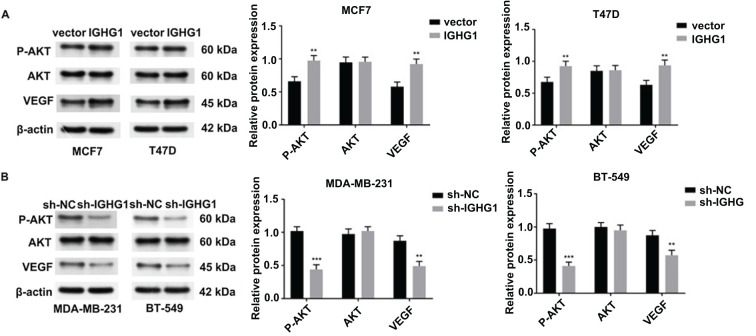
**IGHG1 activates AKT/VEGF signaling.** (A) Protein levels of p-AKT, AKT, and VEGF in vector- and *IGHG1*-transfected MCF7 and T47D cells were detected by western blot; (B) Western blot detection of p-AKT, AKT, and VEGF in sh-NC and sh-*IGHG1* MDA-MB-231 and BT-549 cells. IGHG1: Immunoglobulin heavy constant chain gamma 1; VEGF: Vascular endothelial growth factor.

### AKT and VEGF signaling is upregulated in *IGHG1* overexpressing cells

To dissect the molecular mechanisms governing the effects of IGHG1 on breast cancer cell malignancy, AKT, p-AKT, and VEGF levels were assessed in MCF-7, T47D, MDA-MB-231, and BT-549 BC cell lines. MCF-7 and T47D cells were transfected with empty vector and *IGHG1* constructs and p-AKT and VEGF levels were assessed by western blotting. Total AKT levels were unchanged in both vector- and *IGHG1*-transfected cells (Figure 4A), However, a prominent increase in p-AKT and VEGF levels was observed in *IGHG1*-transfected cells (Figure 4A). In MDA-MB-231 and BT-549 cell lines transfected with empty vector and sh-*IGHG1*#1 construct, AKT levels were unchanged in both vector and sh-*IGHG1* lines (Figure 4B), whilst p-AKT and VEGF levels notably decreased in sh-*IGHG1* cells (Figure 4B). These data confirmed the important role of *IGHG1*-mediated regulation of AKT and VEGF signaling.

### IGHG1 inhibition decreases tumor mass in vivo

**Figure 5.**
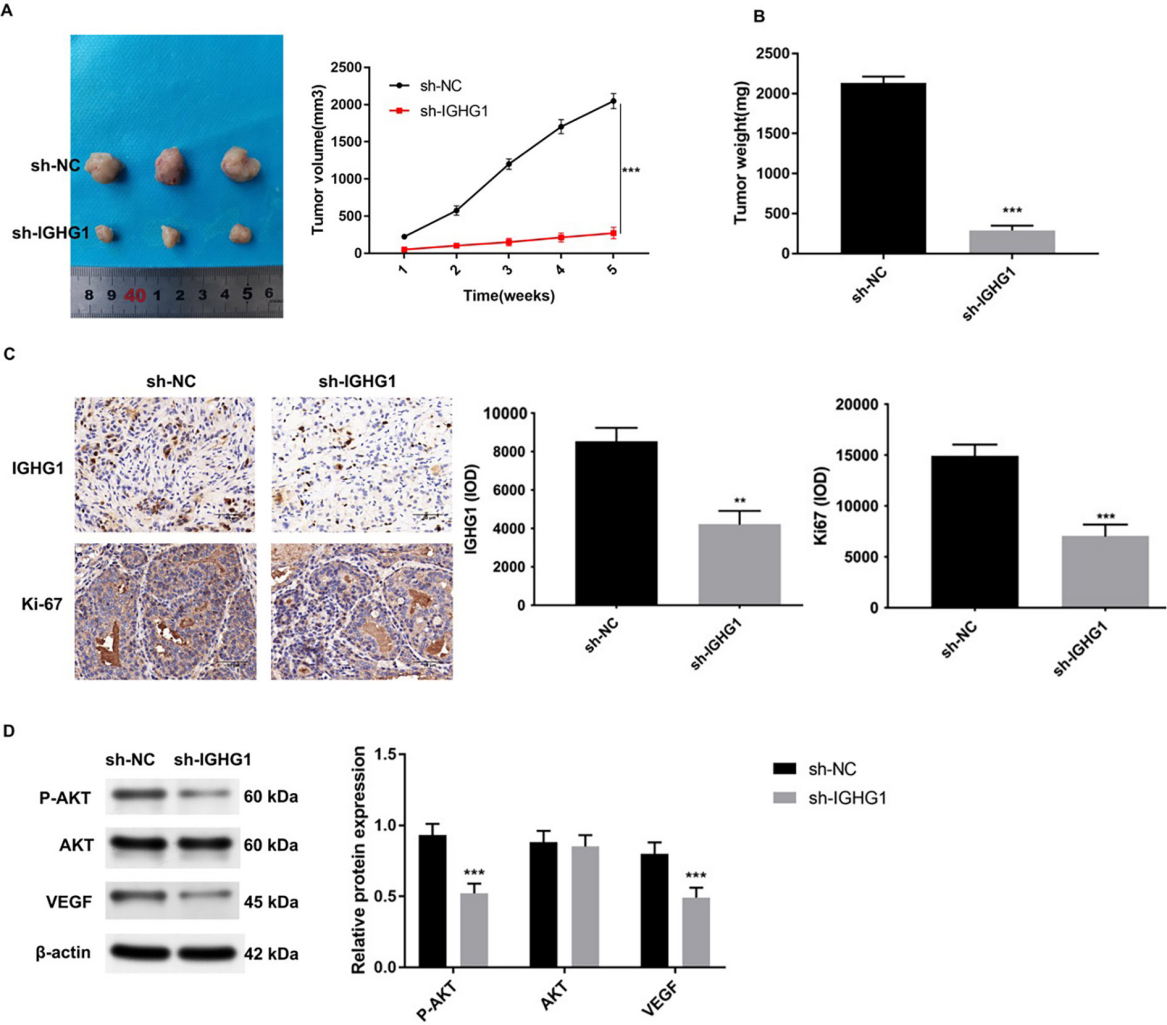
**Knockdown of IGHG1 inhibits the proliferation of breast cancer cells in vivo**. (A) MDA-MB-231 cells (sh-NC and sh-*IGHG1*) were inoculated subcutaneously in nude mice, and tumor sizes were measured every 7 days for up to 35 days. Tumor growth curves were constructed; (B) Subcutaneous tumor masses of the different groups; (C) Immunohistochemistry detection of Ki-67 and IGHG1 expression in subcutaneous tumor tissues; (D) p-AKT, AKT and VEGF levels in subcutaneous tumor tissues of sh-NC and sh-*IGHG1* groups detected by western blot. B-actin was probed as an internal reference. IGHG1: Immunoglobulin heavy constant chain gamma 1; VEGF: Vascular endothelial growth factor.

Athymic nude mice were used to investigate the in vivo effects of IGHG1 on breast cancer cells. MDA-MB-231 cells were inoculated subcutaneously and tumor sizes were monitored every seven days for five weeks (Figure 5A). The control group was injected with MDA-MB-231 transfected with the empty vector. The test group received cells transfected with the sh-*IGHG1* vector. A 6-fold reduction in tumor size and weights were observed in the *IGHG1*-knockdown group (Figure 5B). The tumor proliferation marker, Ki-67, and IGHG1 levels were assessed by hematoxylin eosin (H&E) staining and IHC (Figure 5C). Fewer Ki-67 positive foci in sh-*IGHG1* treated cells were observed compared to the control group. IGHG1 expression levels were also lower in the sh-*IGHG1* group.

Western blot analysis of the control and sh-*IGHG1* groups revealed no changes in total AKT levels, but a significant reduction in p-AKT (Figure 5D). VEGF levels were also lower in the *IGHG1*-knockdown group.

## Discussion

Through TCGA analysis, qRT-PCR, IHC, and western blot, we have confirmed that IGHG1 expression is upregulated in breast cancer cells. These data are consistent with the accumulation of immunoglobulin heavy chains and IgG in malignant breast tissue [15, 16]. The purpose of this study was to validate the association of IGHG1 expression with breast cancer signaling pathways in IGHG1 overexpressed cells. One study has demonstrated that high expression of IGHG1 enhanced the breast cancer cell viability, proliferation, and invasion, but reduced cell apoptosis through modulating the AKT pathway [17]. We have demonstrated that IGHG1 overexpression promotes cell proliferation, angiogenesis, and metastasis in breast cancer cells, consistent with its role in prostate, gastric, and colorectal cancers [18].

Using *IGHG1*-knockdowns in breast cancer cells, we further show that *IGHG1* inhibition suppresses the neoplastic characteristics of breast cancer cell lines. An in vivo breast cancer model established by the subcutaneous inoculation of MDA-MB-123 cells as a xenograft in nude mice also demonstrated tumor regression in *IGHG1*-silenced cells consistent with previous studies in prostate [18], colorectal, and gastric cancers [9, 19].

The role of IGHG1 in various malignancies is now established [10, 16, 20], but the underlying mechanisms and pathways have not yet been discovered. In prostate and colorectal cancer, MEK/ERK/c-Myc signaling is associated with IGHG1 overexpression [16]. In gastric cancer, AKT/GSK-3β/β-catenin signaling is upregulated in IGHG1 cells [9]. In this study, we demonstrate the association of IGHG1 with p-AKT and VEGF levels. A substantial increase in p-AKT and VEGF proteins was observed in breast cancer cells with elevated IGHG1 expression. Moreover, the inhibition of IGHG1 led to a significant reduction in p-AKT and VEGF levels [13].

## Conclusion

Our in vitro and in vivo analyses suggest that the IGHG1 represents a suitable prognostic marker for breast cancer patients. Further multi-centered studies are now required to confirm the role of IGHG1 inhibition as a prospective therapy for associated malignancies.
